# The Disparate Approaches of General Practitioners to the Pharmaceuticalisation of Cardiovascular Disease Prevention

**DOI:** 10.3389/fsoc.2021.650997

**Published:** 2021-05-21

**Authors:** Tom Douglass, Michael Calnan

**Affiliations:** ^1^ Department of Communication and Media, Ulster University, Newtownabbey, United Kingdom; ^2^ School of Social Policy, Sociology and Social Policy, University of Kent, Canterbury, United Kingdom

**Keywords:** pharmaceuticalisation, statins, cardiovascular disease, clinical practice guidelines, general practitioners

## Abstract

In the context of current clinical practice guidance, this paper will analyse the role of GPs in decision-making about the primary prevention of cardiovascular disease (CVD) using the concept of pharmaceuticalisation. Drawing on thematic analysis of semi-structured interviews with 20 GPs, the paper argues that the way GPs approach CVD pharmaceuticalisation is shaped by their understandings of and use of guidelines (and the knowledge they embody), existing treatment perspectives and the moral qualities of preventative treatment, and professional evaluations of ‘relevant’ information. The analysis indicates that there exist disparate and distinct approaches to and understandings of CVD pharmaceuticalisation amongst GPs. Depending on how knowledge, treatment perspectives and values variously combine, GPs sit somewhere on a spectrum of how pharmaceuticalised they are in terms of the approaches to and understandings of the prevention of CVD.

## Introduction

Under current clinical practice guidance known as CG181 (NICE, 2014a), produced by the National Institute for Health and Care Excellence (NICE), general practitioners (GPs) in England are directed to offer advice on prophylactic measures when a patient has a ≥10% risk over ten years of developing cardiovascular disease. Importantly, this includes offering the class of pharmaceuticals named HMG-CoA reductase inhibitors, or as they are more widely known, statins, to try to prevent CVD. The drugs aim to reduce low-density lipoprotein (LDL) cholesterol (‘bad cholesterol’), an important risk factor for CVD. However, risk calculation also takes into account sex, family history, ethnicity, modifiable lifestyle risk factors such as smoking, blood pressure, and geographical factors such as whether an individual lives in the north or south of England (National Clinical Guideline Centre (NCGC), 2014: 5). These risk factors are in part bound up together. For example, modifiable lifestyle risk factors contribute to cholesterol levels in most cases of high cholesterol, and broadly statins can be thought of as ‘risk reducers’ that are prescribed because of overall risk calculation and strategy. When looking at these factors, if the risk profile of an individual is 10% over ten years, this means that in the next ten years from a group of 100 similar people ten would have, for example, a heart attack or stroke. However, if all of those 100 took a statin four of those ten would be prevented from having that heart attack or stroke (NICE, 2014b).

The use of these drugs in the primary prevention of CVD has been viewed in some professional and popular discourse as controversial. As what is considered unacceptable levels of risk have expanded, there has been debate about benefit and safety in medical circles. Indeed, there was a high-profile medical dispute, covered widely by national British newspapers in 2014, when the most recent NICE clinical practice guidance (CG181) was published. This latest guidance halved the risk threshold from ≥20 to ≥10% risk over ten years, making millions more people eligible for statins. This guidance ran contrary to the fact that some other analysis suggests that the safety and efficacy of statins may be questionable at lower levels of risk (see Abramson et al., 2013). Indeed, there have been concerns about pharmaceutical industry funding of research forming the evidence base and hidden trial data (Godlee, 2016)—which are well-established criticisms of the industry generally (Lexchin et al., 2003). Additionally, there were concerns that those on NICE’s purportedly independent Guideline Development Group (GDG) might have links to the pharmaceutical industry (Wise, 2014). Concerns surrounding promoting healthy lifestyles, or what might be termed lifestylism (Hansen and Easthope, 2007), which refers to the emphasis on the individual responsibility for ensuring health through the adoption of ‘healthy’ lifestyle practices (e.g., diet, exercise, smoking cessation), also encircled the guideline’s publication—notions which exist uneasily alongside widening deployment of statins to lower thresholds of risk (Polak, 2017). Though lifestyle recommendations were also made in CG181, with NICE suggesting that lifestyle risk factors should be modified alongside and/or prior to the initiation of a statin, the centrality of statins to NICE’s recommendation was problematised in both professional circles and in the mass media on the grounds that it did not do enough to challenge unhealthy lifestyle practices. The controversy was so significant that NICE were forced to abandon attempts to include patients with a calculated risk within the new primary prevention risk threshold in the Quality and Outcomes Framework (QOF). This meant that GPs (at the time of the data collection for this research) were not monitored or incentivised to prescribe statins to these new patients in the same way as patients with higher levels of risk.

It is worth noting that this case exists in a broader context of debate and controversy about evidence based medicine (EBM). The EBM movement emerged in earnest in the 1990s, attempting to make medical practice more scientific (and cost-effective) by grounding it in randomised controlled trial (RCT) evidence rather than more idiosyncratic experience or tradition. This approach assumes that valid evidence is obtained from expertly produced and accumulated research rooted in a hierarchy of evidence (with RCTs the gold standard). The model tends to view GPs as too busy to access and/or insufficiently skilled to interpret and use this best available evidence in their practice. As such, this knowledge is disseminated to them through guidelines, with professional practice expected to be subsequently shaped/modified in line with the guidance (Harrison et al., 2002: 4–6). Though EBM is now dominant, there are general concerns, argue Greenhalgh et al. (2014), that vested interests distort evidence, that the amount of evidence (particularly in the form of clinical practice guidelines) is now intractable, that increasing focus on statistical significance and risk of disease can mean that gains are marginal, and that guidelines do not always map well onto the individual clinical consultation and crowd out clinical expertise to the detriment of shared decision-making and patient care. ‘Real’ EBM, argue these authors, actually involves individualising evidence for the patient, is based on judgement rather than strict adherence to guidelines, is rooted in strong interpersonal relationships between patients and clinicians, and across these aspects, centres on ethical judgements and shared decision-making. There are clear tensions then, between the RCT model of EBM, which NICE guidelines embody, and other approaches which valorise holistic and individualised medical care.

In the context of the latest clinical practice guidance produced by NICE, this paper analyses the perspectives and understandings of GPs about the prophylactic treatment of CVD. It focuses particularly on the approaches of GPs to preventative treatment in the newer category of risk when a patient is established to have between 10 and 19% risk over ten years of developing CVD. It draws on the conceptual and empirical dimensions of pharmaceuticalisation to evaluate the extent to which GPs display a pharmaceuticalised approach. The paper overall finds a stark disparateness between GPs in understandings of and approaches to CVD prevention. Before presenting the analysis of the data, the paper sets out the conceptual approach and reviews the empirical literature on and relevant to CVD pharmaceuticalisation in the next two subsections.

### Pharmaceuticalisation

How can the prescribing practices of GPs in the above context be analysed sociologically? In this section, the paper sets out the conceptual and analytical framework the paper draws on. Pharmaceuticalisation is a sociological process that is defined by Williams et al. (2011: 711) as the “transformation of human conditions, capacities or capabilities into opportunities for pharmaceutical intervention”. Pharmaceuticalisation relates to and builds on the older concept of medicalisation—which is a broader process whereby social problems come to be defined and treated as medical conditions (Conrad, 2007). It is possible for pharmaceuticalisation to occur with or without medicalisation. Unmedicalised forms of pharmaceuticalisation occur largely outside of traditional medical spheres and/or without any change in or application of a new diagnostic category. Drugs, such as Adderall for example, have been shown to be used beyond their intended medical usage (to treat Attention Deficit Hyperactivity Disorder) and outside of prescription for the purposes of cognitive enhancement and stimulation (Vrecko, 2015; see also; Coveney et al., 2011). Medical professionals, medical settings and diagnosis are excluded from unmedicalised forms of pharmaceuticalisation. This appears to be of greater interest to Williams et al. (2011), reflecting the broader distinction between pharmaceuticalisation of this type and the concept of medicalisation (and thus the uniqueness of pharmaceuticalisation) (Williams et al., 2017).

However, it is important to be clear that these authors do not only limit their conceptualisation of pharmaceuticalisation to this focus. They discuss how pharmaceuticalisation can occur beyond traditional medical spheres, but also within. The focus on pharmaceuticals in and of themselves as analytic entities is a significant contribution of pharmaceuticalisation across both medicalised and unmedicalised forms (Coveney et al., 2011: 387; Williams et al., 2017). In this regard, these same authors have more recently argued that the “value of pharmaceuticalisation… is not solely to do with those instances which extent beyond medicalisation but also those occurring *within* medicalisation” (Coveney et al., 2019: 269). Considering the scale of the use of pharmaceuticals prescribed by medical professionals, which in England includes 48% of the population taking at least one prescribed medicine in the last week (Moody and Mindell, 2017), the utility of the concept of pharmaceuticalisation is clearly far greater if it can incorporate pharmaceuticalisation within as well as beyond traditional medical spheres.

Where medicalisation and pharmaceuticalisation occur together, pharmaceuticalisation is occurring within traditional medical spheres and/or where new or widening diagnostic categories are interlinked with widening pharmaceutical deployment. However, where a more medicalised form of pharmaceuticalisation appears to be occurring, utilising the conceptual dynamics of pharmaceuticalisation to analyse the case rather than medicalisation will reflect the primacy of pharmaceuticals within diagnostic transitions, decision-making or even where developments surrounding pharmaceuticals can be shown potentially to drive medicalisation (e.g., through industry attempts to ‘sell sickness’ and create conditions that can be treated using pharmaceuticals). When drugs are central analytic entities, even where medicalisation is also occurring, the greater intensity and specificity of focus of pharmaceuticalisation on pharmaceuticals themselves and the actors which shape their availability, deployment or consumption means that pharmaceuticalisation is the stronger framework (Coveney et al., 2011: 387; Williams et al., 2017).

This discussion should show the reader how pharmaceuticalisation can occur in or outside of the clinical encounter, alongside or beyond medicalisation. In contrast to instances of pharmaceuticalisation occurring without medicalisation, the expansion of pharmaceuticalisation in the primary prevention of CVD is defined in terms of and facilitated/necessitated by biomedical risk testing. Medical professionals are also the gatekeeper to accessing the treatment. This necessarily means that any subsequent drug treatment occurs in and from medical settings and interactions. Alongside the potential for the deployment of statins, CVD prevention also includes advice about lifestyle change, such as advice on exercise, diet, smoking, potentially in the place of a statin. However, this paper uses the conceptual and empirical dimensions of pharmaceuticalisation because, in the prevention of CVD, once a patient’s level of risk has been established, the central and most salient decision to be made is about whether to initiate statins—or, in other words, whether the patient is to become pharmaceuticalised or not and how this occurs. As will be explored more in the next section, this paper examines how the composition of different knowledge, treatment perspectives, and values possessed by GPs shapes the extent to which they have a pharmaceuticalised approach to CVD prevention that they then bring to consultations with patients.

### Reviewing the Literature

This subsection now turns to review the empirical research conducted on pharmaceuticalisation, with a specific focus on CVD pharmaceuticalisation. There is a vast body of empirical research exploring the six potential empirical dimensions of pharmaceuticalisation set out by Williams et al. (2011). These are the redefinition and construction of health problems as a having a pharmaceutical solution; pharmaceutical regulation; the role of media; patients, consumers, and identities; the use of drugs for non-medical and enhancement purposes; and pharmaceutical futures (see Gabe et al., 2015). These six dimensions can be viewed as delineating a variety of actors and subprocesses that operate as part of a broader network known as the ‘pharmaceutical regime’ and as providing criteria upon which to judge which actors are driving (or indeed preventing/constraining/provoking resistance to) pharmaceuticalisation and its extent.

The empirical interests of this paper relate particularly to the fourth dimension of pharmaceuticalisation pertaining to patients, consumers, identities, and the micro-level deployment and use of pharmaceuticals. This dimension draws on the long history within medical sociology concerned with providing voice to lay understandings and experiences (Britten, 1996; Calnan, 1987). It encourages analysis of patient and particularly consumer identities, understandings and use of pharmaceuticals (Dew et al., 2015). Both in (but also beyond) the study of pharmaceuticalisation, there is a large body of research on the understandings of and decision-making by patients specifically about statins (and subsequent treatment pathways) and associated understandings of cholesterol and risk assessment (Culpit et al., 2020; Farrimond et al., 2010; Gale et al., 2011; Jovanovic, 2014; Polak, 2016; Saukko et al., 2012). Polak (2017), for example, notes the complex web of norms and morals that individuals balance in deciding whether to become pharmaceuticalised through analysing identity construction and the morality of taking statins.

However, as a prescribed medicine doctors have long been important in decision-making about statins^1^. They are, in other words, necessarily an important part in whether a patient becomes pharmaceuticalised. However, research concerned with pharmaceuticalisation has yet failed to analyse the different ways that professionals might understand or approach discussions with patients about statins and the potential impacts of this on pharmaceuticalisation. Consumerist behaviour amongst patients seems particularly limited in the context of preventative medicine (where a patient is ‘at risk’ rather than presently unwell) (Will and Weiner, 2015). Broader work also suggests continuing trust, albeit conditional in nature, by patients in professionals (Calnan and Rowe, 2008). In these respects, it is necessary to attend to professional approaches to and understandings of pharmaceuticalisation and how they interact with patients and subsequently shape pharmaceuticalisation. Whilst Williams et al. (2011) do acknowledge that professional expertise retains a certain level of importance in the decision-making of patients, this dimension, however, is primarily oriented towards patients as consumers in the process of pharmaceuticalisation. Consequently, within pharmaceuticalisation studies less is known about other subprocesses and actors involved in decision-making about drugs and how this might shape (medicalised) pharmaceuticalisation. The role of professional expertise in decision-making about drugs, including statins, requires empirical attention.

GPs’ work is discursively informed by a range of knowledge, values, and multiple understandings of and approaches to pharmaceutical treatments that GPs possess and bring into consultations with patients. In this regard, and in the context specifically of CVD prevention, GPs seem to hold different understandings of ‘appropriate’ levels of risk in primary prevention. Gale et al. (2011), have suggested that, even in relation to prior guidelines and risk thresholds, GPs differed in their understandings of the appropriate level of risk at which to initiate preventative treatment. The guideline at this time suggested ≥20% but Gale et al., show how some GPs preferred to start at 15% and others at 30%. GPs in other research have also seemingly varied in perspective on the appropriateness of the level of risk (Barfoed et al., 2015). In addition, Pollock and Jones (2015) have argued that to appropriately understand and position CVD pharmaceuticalisation it must be analysed relative to other therapeutic pathways that can be adopted instead or alongside pharmaceuticals (particularly lifestyle change). However, how doctors understand and approach the relative benefits of the therapeutic landscape has not been analysed in relation to pharmaceuticalisation. This said, Manca (2018) is one of few examples of pharmaceuticalisation scholarship engaging with professional understandings, narratives, and approaches. This research suggests different orientations amongst and a certain degree of anxiety by some medical professionals towards pharmaceuticalisation, particularly where public uncertainty and controversy exists, and the role of the pharmaceutical industry generally in the production of knowledge. How these aspects might relate specifically to CVD pharmaceuticalisation is unclear, however.

This paper examines how the composition of different knowledge, treatment perspectives, and values as possessed by GPs shapes the extent to which they have a pharmaceuticalised approach to CVD prevention. Reflecting these notions, the research question at the centre of this paper is: how do GPs understand the ≥10% primary prevention category and the utility of statins, and what shapes if and how have they have been implementing guidance about this level of risk?

## Method

### Qualitative Interviewing

The data analysed in this paper is from 20 semi-structured interviews with GPs collected in late 2015 and through 2016. In-depth qualitative interviewing is perhaps the most widely used method in qualitative research and is in the most basic sense, a conversation oriented at exploring an individual’s account of social phenomena (Green and Thorogood, 2004: 79–81). The emphasis in in-depth qualitative interviewing is on the interviewee’s perspective and their account of lived experience (Seidman, 2006: 9–10). This allows for rich detailed answers and descriptions that offer crucial insight into what the participant views as important about the social phenomenon under study. These aspects mean that qualitative interviewing was an appropriate method to use to explore the perspectives and understandings of GPs about CVD prevention, their lived experience of the implementation of the ≥10% primary prevention risk category and approaches to decision-making about statins.

It is important to note that the interview data represents what GPs say about how they approach the prescription of statins, rather than necessarily what they do in practice. Debates exist about the kinds of knowledge claims that can be made from this kind of interview data. It has been suggested that data derived from qualitative interviewing provides idealised accounts that are not necessarily relatable to what a person actually believes or how they behave (Murphy et al., 1998: 105). Equally there is an issue of whether meanings presented in an interview are ever stable and in some way representative of a verifiable internal (perspectives, beliefs) or external (actions taken) reality (Dingwall, 1997: 38; Murphy and Dingwall, 2003). Interviews are thus seen by some as context specific performances involving a degree of impression management. Others, however, suggest that accounts by interviewees can meaningfully be both resource *for* analysis and topic *of* analysis (Hammersley and Atkinson, 2007: 97–98). In terms of the aims of this paper, the researchers were interested in the understandings and perspectives of GPs, rather than the actualities of clinical consultations (as a way to begin to address the gap in knowledge about pharmaceuticalisation relating to the role of medical professionals). This paper, as such, does not wish to infer that what GPs say they do or say they believe can be said to map necessarily onto what they *actually do* in practice.

The decision to use semi-structured interviewing rather than unstructured interviewing reflects the conceptual foundations of the project and the specific dimension of pharmaceuticalisation and associated research question the research team wished to analyse. In this regard, the research team already had a set of topics that were important to address. As with Coveney, 2010 (91–92) there was concern also that using a completely unstructured approach might mean neglecting certain topics of salience to understanding the dimension of pharmaceuticalisation under study. Semi-structured interviewing, however, also afforded the interview process a degree of flexibility to explore further topics of interest as they emerged, allowing participants the chance to present what they believed to be important. The semi-structured interviews were conducted by the lead author using a topic guide. All participants signed a consent form prior to the interview taking place which was used to assert that they were aware of the nature of the research (based on a participant information sheet), right to withdraw, consent to audio recording, and the way data derived from the interview would be used. All interviews were anonymised during the transcription phase (meaning that information that might lead to identification was removed/redacted and every name and location were anonymised). The interview data was then analysed thematically using Braun and Clarke’s (2006) six phase model. Coding and theme formation were conducted primarily by the lead author, but this was discussed with and agreed by the second author. A third researcher also examined coding and theme formation, thus enhancing the internal reliability of the project by building in an inter-observer consistency. The analysis of GP data was guided by interest in the ways in which GPs understand the latest NICE CVD guideline and statins, as well as how they approach, understand, and report contributing to the decision-making of patients. Though theoretically and empirically pharmaceuticalisation has neglected the role of medical professionals, wider theorising and empirical work suggests that medical professionals must be brought back into focus to understand decision-making surrounding drugs more fully, particularly because such decision-making is a shared activity. Ultimately three overarching themes were established. These themes were also further honed to include subthemes with the primary goal of increasing clarity and particularly allowing for emphasis on distinct thematic positions apparent in the talk of GPs to emerge.

### Ethics, Access and Sampling

Relevant ethical and research governance approvals for a broader project that this paper forms part of was granted in 2015 by the University of Kent and the NHS. Following this, access to GP interviewees for the study was negotiated in one Clinical Commissioning Group (CCG) in the south of England. Whilst it might have improved the external validity of the research (as well as potentially adding a comparative element) to have collected data in two or more CCGs, evidence also exists to suggest that GPs are heterogenous and individualistic even in the age of clinical practice guidelines (Armstrong, 2002; Spyridonidis and Calnan, 2011). A relationship was established with a member of the CCG’s board who displayed particular interest in the topic. This board member, who was also a GP, acted as a gatekeeper to accessing GP interview participants. An email advertisement was sent to every GP working within the CCG. The response was initially muted, but several further emails were sent over a period of nine months (in line with ethical approval). A purposive sampling approach was taken. At the most basic level, qualification to participate was based on being a GP currently working within the CCG with an aim of recruiting an even split of male and female GPs. In particular, the researcher set out to establish a sample diverse in terms of levels of clinical experience. The rationale here was that differences in clinical experience might shape how GPs approached the use of guidelines (due to the clash between clinical experience and guidelines noted in other research discussed above). A sampling frame was established relating to years of clinical experience (early or later career), with the aim of interviewing between seven and ten GPs with ten or less years clinical experience as a GP. The sampling also ultimately benefitted from elements of snowballing with GPs on several occasions recommending and asking particularly younger colleagues if they would consider participating. Ultimately twenty GPs were interviewed. Twelve were female and eight were male, with seven having ten or less years of experience.

## Results

The thematic analysis of the interviews with GPs is presented in three overarching themes: Use of Guidelines, Treatment Perspectives, and Evaluations of Relevant Information. As such, the analysis begins with an examination of the manner in which GPs perceive and use guidelines and knowledge generated and disseminated via EBM particularly compared to emphasis placed on the importance of their own clinical experience and discretion. The paper then examines how this relates to how GPs reported they were implementing relevant components of CG181. It then discusses differential perspectives on and understandings of treatment. This is analytically distinct from the first theme in that it captures differing perspectives and preferences about treatment options as relates to, for example, the moral aspects present in the talk. The final theme presented in the paper highlights how ethical and personalised patient evaluations feature in the presentation of ‘relevant’ information to patients. It is argued overall that disparate approaches by GPs to CVD pharmaceuticalisation across these themes can be identified.

## Use of Guidelines

In this first theme, the paper focuses on how GPs perceive and approach the use of NICE guidelines, and the specific form of knowledge it embodies. From the data analysis, it very clearly emerges that the ways in which GPs engage with the ≥10% primary prevention risk category reflects existing understandings of and engagements with EBM. The analysis is divided into two thematic positions.

### Positive about NICE Guidance

The first thematic position that emerged from analysis of the data pertaining to GP perspectives on NICE guidelines was of acceptance of and need to conform practice to EBM in medical practice. This position reflects the perceptions primarily of GPs who were judged to have spoken positively about the role of NICE guidance in contemporary primary care. GPs within this thematic position here can be thought of as accepting of the logic of EBM. NICE guidelines, though as part of a broader decision-making process, were seen by GPs here as the “gold standard” (GP3) and the best available and should be followed (where appropriate).

Acceptance of the logic of EBM, and as such, NICE guidelines more specifically, was rooted in several aspects.

We are working in a very broad sense. We are gatekeepers, we never have a clue what is coming through the door and we have to know a little bit about everything. I think that is actually quite difficult… If we didn’t have some guidance in certain areas to follow it would be quite tricky to have a uniform approach to things (GP13).

This quote from GP13, a younger GP with a lower number of years of experience, suggests that NICE guidelines are necessary because of the nature of general practice and the need for uniformity in practice. This is echoed by GP5, this time a very experienced GP, who stresses how difficult general practice might be and specifically managing voluminous evidence.

I am thoroughly in favour of NICE and I am very glad about their existence; and I think it would be very difficult to practice with the huge amount of evidence in modern medicine, the huge wealth of data, if you didn’t have NICE there to do it. So yes thoroughly in favour of NICE guidance (GP5).

The importance of uniformity was also stressed by GP6, an experienced and older GP, with an emphasis this time on protecting patients from dangerous practice.

I see it as trying to make sense of a sophisticated modern health service and trying to make sure that nobody is doing anything that sort of are crazy or even dangerous. The evidence these guidelines are based on is the best evidence we have so we should try to follow it (GP6).

Trust in NICE by GPs taking this position was also important. GP7, a relatively young and less experienced GP for example, trusted NICE as ‘the experts’ as a way to navigate (humble) concerns about their own lack of speciality and related ability to keep up with and make sense of the underpinning or developing research evidence.

As a GP I don’t think I have any particular expertise and I have a lot of faith in the people who develop the guidelines… as having more knowledge about a particular topic than I do, do you know I want mean? So you know, whilst it is quite nice to have free range to do whatever you like, I’m happy that the people who develop the guidelines are in inverted commas experts and as such they are generating good advice if that makes sense (GP7).

GPs who spoke in this way were also generally positive about the latest primary prevention category and the widened availability of statins because of this. GP3, for example, one of the least clinically experienced GPs in the sample, felt that targeting patients at lower levels of risk would get people on a statin earlier who would need one eventually anyway.

I mean somebody who has got a 20% risk and had it for a few years is likely to have really furred up arteries. If you can get them on a statin at [lower] risk a couple of years earlier, then that can only be beneficial (GP3).

More specifically, the population level approach necessitated in the treatment of risk of CVD (and indicative of EBM due to being rooted in RCT data and disseminated by guidelines) was positively viewed. This kind of GP saw the change as having potentially beneficial impacts on the levels of cardiovascular events in the population and which necessarily shaped considerations of benefit even in individual consultations. This sentiment was clearly articulated by GP18, also younger and with only a few years of clinical experience, who compared statins to vaccines because they have population level benefit (rather than solely or even primarily, an individual benefit).

My outlook on the usefulness of these [drugs]… does include consideration of population level benefits. And I suppose for me statins are like vaccines to a certain extent (GP18).

In addition, though concerns about the adverse effects of statins had featured prominently in professional and popular discourse surrounding this guideline, these GPs (though aware of these debates) generally saw statins to be a safe drug, largely reflecting their views of the underpinning evidence and the logic of EBM. GP12, this time an experienced GP, here argues that statins are safe drugs and that people convinced statins were harming them had increased likelihood of CVD morbidity, despite contrary evidence.

I’ve seen significant morbidity from people who are convinced that statins are doing them harm. I think illogically. Very occasionally you see significant harm from a statin, but very rarely in my experience and that is supported by the available evidence that we have (GP12).

### Negative about NICE Guidance

However, other GPs stressed a greater emphasis on the negative aspects of NICE guidance. Important here were notions that NICE guidelines were dictum from above and actively devalued or had the potential (if a doctor too rigidly attempted to follow them) to devalue individual discretion and clinical autonomy. This was used as a basis to criticise the value of EBM. GP8, a very experienced GP, for example, spoke of professional experience as like an additional sense, but that this was being devalued by the mechanisms of EBM.

…training and clinical experience has got to count for something hasn’t it? Increasingly the experience side of it is being devalued. Erm before long they will be training monkeys to do our job, or certainly machines. If you take the sort of sixth sense out of it then you know what are we there for? (GP8).

Due to this devaluing of clinical experience, EBM as embodied by guidelines was for this type of GP of limited acceptability. One of the most experienced GPs, GP1, also emphasised, for example, the importance of professional experience and knowledge in terms of the quality-of-care patients receive.

… my personal view is that the particular and so-called evidence-based medicine on which guidance is based has limited value in that I think that evidence-based decision making should take into account evidence-based medicine in the sense of NICE, but also the patients’ wishes… but also long term clinical experience and professional knowledge (GP1).

It was also suggested that NICE guidelines did not necessarily translate well into real world consultations and were created with some sort of ideal patient in mind rather than the one in front of them. GP8, for example, directly challenges this and also articulates the fear of and resentment about potentially being sued for not following them.

I do not necessarily think that they are always in the individual patient’s benefit. Erm so I will discuss it with the individual patients but we won’t necessarily adhere to them… It’s not so much that I object to their use, what I object to is the one size fits all…Erm so you know if they were just guidelines it would be fine but they sort of but they are marketed as very dogmatic rules that could lead to us being sued (GP8).

GP1 meanwhile suggested there might be reasons for not trusting guidelines and those that produce them due to evidence not being available (seemingly echoing well known concerns about the influences of commercial influences on what data is shared and published).

I think they [NICE] are probably responding to research evidence here—but only some of the research is published and available (GP1)

This GP continues by rejecting the level of risk justifying the intervention, and as such, the population level logic for prescribing statins to a wider number of people as recommended by the guideline.

I think in this case it is a [question] of at what point they are recommended. And actually whether we are treating a lot of people unnecessarily to reduce one event. So the case of numbers needed to treat…if we are having to treat 200 with the view of reducing one cardiovascular event or separate vascular incidents, you know a lot of people get side effects with statins therefore it would be questionable at this level… I think I’d have to see a stronger argument for benefit before subjecting patients to likely risk of side effects. … I think we are broadly beginning to fiddle around the edges of benefit. Kind of, we have tackled the low hanging fruit (GP1).

In the second half of this extract, it is also clear that this GP problematises the likelihood of increased instances of side effects for individuals who may gain little from taking the drugs. In this way, this GP seemed to judge their own professional knowledge and experience to be more reliable than the NICE clinical practice guideline. Meanwhile, here GP17, a GP also with many years of clinical experience, discusses how the lower risk threshold, from their perspective, is potentially too low because, again, the risk of side effects do not seem to be justifiable relative to benefits to individual patients. Interestingly, again, there is also a negative perception relayed about the distinction between overarching population risk prevention, and those who create these strategies, and the individual patient sat in front of the GP.

…in my mind the numbers needed to treat kind of balance better at the 20% than they do at the 10%. You have to treat vastly more people to reduce one person from having a heart attack or a stroke. So you know obviously in terms of the population as a whole you know if you are a strategist, a health strategist you see this as a very viable sensible thing….But as a GP you kind of think ‘ughhh’ when you know that a good 20% do get statins side effects, whether they recognise them or not—in terms of fatigue, energy levels, ability to think, muscle aches and so on and so forth (GP17).

For this kind of GP then, in contrast with their colleagues above, there was a clear clash between what guidelines intended (operating with population level focus) and the real patient sat in front of them. The most salient point here is that guidelines were thought to devalue professional discretion, experience, and knowledge, but due to the individualised experiences and needs of patients, these aspects were in fact perceived as inherent necessities. There was evidence of negative perceptions about those who create guidelines when compared to the realities of consultations with patients and the work done by GPs.

Only one GP within the sample had been completely ignoring the lowered primary prevention threshold—and thus totally resisting the pharmaceuticalisation of patients. However, other GPs with critical attitudes towards guidelines had been discussing risk testing and reduction with patients who had a risk level between 10 and 19% mostly because they felt legally pressured to record they had done so or (more commonly) duty bound as part of facilitating informed patient decision-making to conduct risk testing and discuss treatments options with patients. As is explored later in the paper, however, there was sometimes emphasis by the type of GP with these perspectives on engaging with lifestyle changes rather than utilising a statin to lower risk, which was partially rooted in dissatisfaction with a population level, collectivised rationale, and the greater individualised benefits from lifestyle changes than from a statin.

## Treatment Perspectives

The analysis of the data also suggested that there were three different perspectives about the therapeutic landscape (statins and/or lifestyle change) in the ≥10% risk category. GPs held disparate opinions, often quite distinct from the specifics of the guideline about the best approach to the treatment of CVD risk. Whilst this partially connects to and reflects the above discussions about use of and approaches to guidelines themselves, there were also aspects that emerged from the data that were distinct from these considerations and stand as important analytical insights on their own—most notably relating to the moral qualities of treatment.

### Statins and Lifestyle Changes Together

Some GPs (the largest group) saw the ≥10% threshold in terms of a unified therapeutic landscape and emphasised the necessity for (typical) patients of making lifestyle changes prior to and subsequently alongside taking a statin. GPs here in this category broadly focused on the preventative benefits that would come from a unified treatment approach. This is exemplified by GP3 describing their approach to consultations with patients.

I frame it as ‘there are some things that will help bring risk down, lifestyle changes, and medications we can offer that will help, including a statin.’...Part of a suite of things that we are trying to do (GP3).

GP18 similarly emphasises how statins can help lower risk but not instead of lifestyle changes, stating that it is not ‘lifestyle versus statins’.

I think, I suppose, I don’t view it as lifestyle versus statins…I will always say, you know, my phrase always goes along the line of well we should be thinking about starting a statin which will help to lower your risk of a heart attack and stroke, but this isn’t instead of making lifestyle changes (GP18).

GP10, a younger and less experienced GP, meanwhile suggests that starting a statin might make people complacent about making lifestyle changes because they think the statin will protect them. There is also a subtle emphasis by GP10 on the moral need for patients to make lifestyle changes rather than relying on a statin, evidenced by the use of the word ‘complacent’.

I think for most people I think it is important that we tackle that [lifestyle] side of things first. Because I think they can be complacent. Some people think if we put then on a statin they think ‘oh that is fine’ (GP10).

Interestingly, as this quote from GP7 indicates, there was a sense of professional responsibility to encourage lifestyle changes (e.g., stopping smoking) rather than only prescribing drugs evident in the talk of this sort of GP.

From my perspective if someone smokes there is hardly any point in someone taking a statin, you know what I mean?… You might make a tiny dent in their risk comparative to what stopping smoking will do… I’m not always saying have a statin, I’m saying lets see what happens if we you know we can stop the smoking (GP7).

### Superiority of Lifestyle Change

Another slightly smaller group of GPs suggested that they actively attempted to promote lifestyle change in place of a statin where risk level was between 10 and 19%. It was not necessarily that these GPs would not offer or prescribe a statin at this threshold (particularly when deferring to a patient centred model of care), but these GPs reported that they advised and emphasised to patients the benefits of lifestyle changes to a much greater extent than they promoted a statin.

This category of GP was more clearly motivated by a moral imperative surrounding looking after one’s own health rather than relying on drugs. This is evident here where GP17 emphasises ‘reliance on tablets’ at the expense of healthy living. This was a GP who, importantly, had spoken (as can be seen in the previous section) quite negatively about the underpinning rationale of the guideline. The connection here between problematising EBM and preferences for lifestyle change can be seen.

Of course, one of the biggest difficulties with [statins], um we have become a nation that relies on tablets…. The risk of statins is that people carry on living the same lifestyle of over-eating of over-drinking, not doing exercise (GP17).

GP20, a very experienced GP, makes a similar point, emphasising that drugs are increasingly seen as the answer to problems of lifestyle.

My problem is that it is just… giving out pills for everything, rather than, you know, it is just that people need to eat better, exercise more (GP20).

GP11, meanwhile, emphasises interestingly (particularly as a GP with only a limited number of years of experience) that while guidelines suggest that patients should take a statin, it was a superior approach to avoid the drugs. This leaves the impression that the unified approach to treatment contained in the guideline is morally inferior to reducing risk via lifestyle changes alone.

I’d say you know there are guidelines that suggest you should be on some medicines now because of the cholesterol and the diet and that kind of thing. But I generally say we want to prevent that if we can (GP11).

### Statins Independently

The smallest group of GPs reported placing significantly more emphasis in their framing to patients on the importance of a statin relative to the benefits from lifestyle change. There was a clear acceptance by GPs in this subset of the ≥10% risk category, its necessity and that it was a risk level that required the application of treatment. And in this way, the drugs were a morally neutral application of the best available evidence. GP6 spoke, for example, about the clear evidence-based benefits of a statin and compared this to their understanding of the evidence based benefits of lifestyle changes.

You see there’s some evidence that even a low dose statin is extremely effective in prevention. So even if a patient is not very tolerant and they are taking a low dose of something they are still getting an awful lot of protection. Erm it’s much less clear, apart from smoking, how much say weight loss, or exercise has an impact. They are complementary but independent of too (GP6).

GP12 here also suggested that the structure of society was set up in such a way that facilitated and perpetuated unhealthy lifestyles, and statins were a necessary (if unfortunate) solution to this. This seemed to partially make patient use of statins morally neutral in the view of this GP, whilst also meaning it was necessary and legitimate for them to prescribe (providing a sort of moral absolution for the GP too).

Well lifestyle choices have a huge impact, do a lot of exercise, doesn’t smoke, healthy lifestyle you know. Then they are much less likely to get cardiovascular disease. But that is influenced so much by culture, structure of society, which is nothing to do with their GP. Lifestyle change is hard to make and keep up. How important is it? Well it is hard to assess actually and to keep up. For a GP… verging on the impossible… Statins, I can do (GP12).

The three positions identified in this theme clearly show distinct approaches to and understandings of the treatment of CVD risk in the 10-19% risk category. In other words, GPs were highly disparate in the extent to which their treatment perspectives were pharmaceuticalised.

## Evaluation of Relevant Information

Though the above theme captures much of importance in terms of the approach preferred by GPs within the context of shared decision-making about the therapeutic landscape at the 10–19% level of risk, further qualifications and complexities emerged in the data. In the data analysed, it was apparent that treatment decision-making in the ≥10% risk category was also reflective of a number of ‘evaluations’ that GPs reported that they conducted prior to and within the context of consultations. These evaluations informed what was considered to be relevant information and its presentation to patients.

### Ethical Evaluations

It was clear that GPs in the sample undertook a variety of evaluations about what was considered relevant information to a patient that can be thought of as ethical evaluations. For example, GP20, who, as seen above, problematised the widened primary prevention category, was concerned about whether a ≥10% primary prevention threshold was an appropriate threshold for intervention and thus should be considered ‘relevant information’ to the patient. In this data extract, GP20 expressed a concern that telling patients about their risk at this threshold would cause patients unnecessary stress, worry, and potentially contradict the medical ethical principle of nonmaleficence (the intentional avoidance of harm).

I’ve found it hard to even talk to patients…. Some of them will worry… why would I want to make them worry when I don’t really believe they are significantly at risk of an event? (GP20).

A further ethical dilemma surrounding evaluation of what constitutes relevant information also emerges in a different and slightly more obscure way—in terms of whether GPs decided to tell patients that the ≥10% category represented (at the time of data collection) a recent change. Indeed, GPs had varying orientations to and rationales for whether they explained to patients that this threshold represented a change in policy. Whilst this may only be a small element of some consultations with patients, what is important here is the disparities between GPs in their approaches again. Differential framing by GPs here means that patients have more or less access to relevant understandings/perspectives/knowledge, which in turn may feature in how they understand risk and therapeutic options. It was seen by some GPs as relevant information, and for others, as something unimportant to patient-centred care and decision-making, despite the controversial nature of the change and the potential pertinence of information, for example, that less than two years previously patients would not have been offered a statin. For some GPs, such as GP2, an experienced GP, a changing guideline was not really seen as relevant to patient-decision making and that patients would not be interested in this fact.

TD: Do patients know that something has changed in terms of NICE guidance?

GP2: I’m not sure they really understand there has been a shift here.

TD: So it’s not part of your conversations?

GP2: I don’t bring that up. I don’t say look it used to be 20% but we’ve brought this down. I just say that look you fall into the threshold where we would normally offer cholesterol lowering medication.

This perspective was mirrored by GP18 who did not think the older guidance remained relevant to patients in the present.

I do not say anything about changing guidelines to them, I suppose I don’t tend to see it as particularly relevant to them what the previous guideline used to say. I think that the current guidelines are the ones that we are following and they are the best practice at the moment (GP18).

For GPs thinking in this way, it was only seen as necessary to explain to patients who had been told during a previous assessment that CVD risk and/or cholesterol were unproblematic, thus causing them to question whether their health had become worse.

For GP13, it was apparent that there was a conscious decision not to talk about the fact that the threshold had changed because they did not want to give patients room to protest against interventions (as seen above, this GP positively viewed NICE guidelines).

I personally only say that if they were someone who previously… told that they were fine… I wouldn’t say that has recently changed. It would just give them more reason to protest (GP13).

Other GPs, such as GP14, an experience GP, however, had more actively discussed the change of threshold with patients—with a suggestion that it was ethically necessary to provide this information.

You know a year ago or however long it was, two years ago we were saying it was 20% and I think that immediately sets a different tone to the conversation really doesn’t it (GP14).

It was clear, as such, in the talk of certain GPs, that by presenting information to patients about the changed threshold, having evaluated that this was necessary, there was an explicit acknowledgement that this information could shape decision-making about statins, and as such, was ethically necessary in terms of making patients aware of surrounding debates. It was also important ethically in that it facilitated informed decision-making in the face of potential patient confusion. Whilst the importance of this element of GP practice is perhaps unlikely to be important in a long-term sense, there was a clear disparity apparent here in GP practice in the 18-24 months between guideline publication and data collection.

### Personalised Evaluations

GPs also placed differential personalised emphasis on particular elements of a patient’s lifestyle considered problematic, such as smoking, as was evaluated as relevant to the individualised context of the patient. Some GPs also seemed to draw on what they knew about the patient’s overall life. GP15 suggested that for many people, including at 10%, it is hard to make lifestyle changes due to various social arrangements and commitments, reflecting the structure of society—thus prompting this GP to offer a statin earlier in the process and more forcefully to patients. For GP15, an experienced GP, there is a clear focus here on what might be achievable for patients based on the particular circumstances of the patient, whilst also filtering this advice through personal perceptions and understandings of treatment, in this case, as a GP who believes strongly in the utility of statins.

I still think that people should try to make lifestyle changes but you’ve also got to be realistic sometimes when they are working long hours or something, and thus I often just say ‘we should really strongly think about a statin because your risk falls within the problematic range’… You can lecture people until you are blue in the face about exercise, diet, whatever, but realistically they are living lives that they are struggling with (GP15).

For patients without additional issues such as comorbidities or a strong family history of CVD, risk level relative to therapeutic pathway was also personalised. In essence, for some GPs in the sample, the closer the patient was to 20% the more likely they were to emphasise the need for a statin, whilst closer to 10% risk they would advise patients with more emphasis on lifestyle change. This is particularly interesting when compared to the actualities of the guideline where there is no distinction made between risk reduction strategies at different risk levels within the overall threshold. For GP13, the way in which emphasis was given to particular therapeutic pathways seemed to reflect, as such, a personalised evaluation of the level of risk.

I definitely do think the figure makes a difference to how I would behave in the consultation… if they were on 19% I would be much more keen to talk seriously with them into a statin. If they were 11% then I would probably have a more half hearted effort (GP13).

GP7 had a similar perspective. This GP reported trying to calculate risk level if patients were successful with lifestyle changes, and if this was still going to be high within the risk threshold then suggested that statins might be initiated earlier in the process. Interestingly GP7 elsewhere suggested a treatment preference for a balance of lifestyle and statins to prevent CVD, which is consistent with the comments here. However, how quickly statins were introduced reflected personalised evaluation of risk.

I tend to look at the numbers…I don’t tend to say to patients I don’t think this will work. I say this is what I think is feasible in terms of lifestyle change, this is what your score will be if professionals happens and it’s up to them. I might say I think that’s reasonable because we might be able to do well here, but if it is still gonna be well above 15 then I tend to say ‘we’ll try to make these changes, but we can start you on a statin if you want to now rather than waiting’ (GP7).

## Discussion

This paper has explored how GPs possess a range of knowledge, values, and multiple understandings of and approaches to pharmaceutical treatments in the context of the latest CVD prevention clinical practice guidance. Thus far, reflecting the way Williams et al. (2011) have constructed their conceptual framework of pharmaceuticalisation, research on their fourth dimension of pharmaceuticalisation has primarily explored the consumerist behaviours and pharmaceutical identities of patients in the process of pharmaceuticalisation (Dew et al., 2015; Will and Weiner, 2015). Consequently, within the pharmaceuticalisation literature less is known about other actors involved in clinically situated decision-making about drugs and how this might shape pharmaceuticalisation. Statins are a drug that need to be prescribed, and where pharmaceuticalisation is occurring it does so in a medicalised form. It is important, as such, to analyse the understandings of and approach to the prescription of statins by those who have the authority to prescribe them. It is true that it cannot be known from the data presented what the impacts are of the different understandings and approaches of GPs across the themes on if patients become pharmaceuticalised. However, it has shown how GPs’ role in decision-making about statins is informed and shaped by a range of knowledge, values, and multiple understandings of and approaches to pharmaceutical treatments that GPs possess and bring into consultations with patients. These aspects have not been analysed in the pharmaceuticalisation literature before. As will be shown below, GPs can be argued to sit somewhere on a spectrum of pharmaceuticalised understandings of and approaches to CVD prevention. Where they sit on this spectrum reflects the combinations or dominance of certain knowledge and its use, treatment perspectives and preferences, and a range of moral and ethical values.

Research suggests that GPs do not receive and utilise knowledge and evidence disseminated through clinical practice guidelines in uniform ways (Carlsen, 2010; Hansen et al., 2016; Spyridonidis and Calnan, 2011). This seems to reflect conceptions of medical professional identity unique to the GP, with a significant emphasis on clinical discretion, experience and autonomy (Carlsen and Norheim, 2005) and a pronounced distinction between themselves and specialists that emphasises patient centred and holistic care (Checkland et al., 2008; Hansen et al., 2016). Clinical experience, and the associated existing cognitive familiarity with, for example, a particular treatment, seem to remain crucial and explains reluctance to follow clinical practice guidelines uncritically. The data analysis in this paper shows similarly that a problematising orientation taken towards NICE guidelines by a minority group of GPs was evident in the data, a subtheme which is broadly in line with sociological literature concerned with the understandings and implementation of clinical practice guidelines (Spyridonidis and Calnan, 2011). A rejection of the collectivised population level logic underpinning NICE guidelines and the preventative use of statins was compatible with a lifestyle focus (and the perception of the supposedly greater individualised benefit) for these GPs. Experience level and number of years in clinical practice was also important here. Greater experience levels generally coalesced with negative perspectives about NICE guidelines and greater comfort in practicing outside of the guideline.

However, a larger group of GPs did see the value of the knowledge produced and disseminated through guidelines, in a way not dissimilar to that articulated by McDonald et al. (2009), and this was reflected in the fact that most of the GPs interviewed had been attempting to implement the guideline. The divide between GPs concerning clinical practice guidelines evident in the data has not necessarily been articulated in the same way in other studies, although when looking at the literatures on professionalism and professional identity overall divergent positions are apparent. The acceptance of the guidance, and thus of the logic of EBM was associated with trust in NICE. The importance of trust for actors other than only patients (and their trust in health professionals) in healthcare emerges here then as indicated by other scholarship (Brown and Calnan, 2016; Douglass and Calnan, 2016). Trust was placed in NICE and their evaluative activity particularly to bridge over aspects of uncertainty and where knowledge constraints were acknowledged/evident.

Overall, differential approaches to and understandings of clinical practice guidelines are important to understandings of pharmaceuticalisation. Certainly, the development of a new guideline that creates an opportunity for pharmaceuticals to be more widely available and used, does not mean that this will be how GPs will interpret and approach the prescription of drugs—or even use/abide by them at all (GP1 had been ignoring the guideline, for example). The type of understandings of and approaches possessed by a GP relating to guidelines are one important aspect when examining the extent to which GPs have a pharmaceuticalised understanding of/approach to CVD prevention.

Pollock and Jones (2015) have argued that the analysis of CVD pharmaceuticalisation must be contextualised relative to the broader therapeutic landscape. Sufficiently appreciating this point necessarily means engaging with the cultural context within which decision-making about drugs is taking place (Gabe, 1990)—particularly surrounding notions of individual responsibility for health. In the data, GPs had different understandings of the moral qualities of the drugs and of lifestyle change as treatment options. At the centre of the issue here was the potentially ‘unnecessary’ utilisation of pharmaceuticals where healthy lifestyle practices could be adopted to mitigate risk and a sense of their own professional responsibility to encourage healthy lifestyles. For a number of GPs there was a pronounced narrative of lifestylism similar to that described by Hansen and Easthope (2007) which diminished the desire to pharmaceuticalise patients. Some of these GPs described themselves, as such, as preferring to medicalise participants through discussions of biomedically informed advice about lifestyle changes to improve health. Where GPs viewed statins as morally neutral, less or no medicalisation of this type was preferred. Interestingly then, where GPs described a preference for increased medicalisation or pharmaceuticalisation this was often at the expense of the other process. Polak (2017) meanwhile highlights the complex moral positions patients take on statins use. Similarly, the findings presented above confirm that GPs also possess disparate understandings that reflect moral positions pertaining to perceptions of individual responsibility for health that influence treatment perspectives. GPs themselves, of course, do not exist in a social vacuum and the line between medical knowledge and social emphasis on individual responsibility is blurred (Hansen and Easthope, 2007). The different positions of GPs are important to appreciate though because GPs’ perspectives (at least as far as they allow them to emerge in consultations) are likely to be one important aspect in the configuration of patients’ own moral positioning on treatment—although this requires further empirical exploration. Certainly, again, that GPs have their own moral perspectives on different treatment pathways is important for sociological understanding of pharmaceuticalisation. Whilst guidelines (embodying EBM) are supposedly the neutral dissemination of the best available evidence, GPs’ clearly bring a range of perspectives to the decision-making process that may contradict what guidelines recommend. This has not been noted in the pharmaceuticalisation literature before now.

It is also clear that decision-making about statins in the ≥10% risk category is discursively informed by a range of other values that GPs possess and bring into consultations with patients. Clinch and Benson (2013) show how GPs attempt to order and present information to patients in ways that related to the specific circumstances of the patient. Similar aspects were found in the data analysed above—for example, as related to individual patient risk level within the broader risk threshold. This data also mirrors older research that has shown that doctors in the context of older guidelines varied in the level of risk that they felt was sufficient to begin interventions to prevent CVD (Gale et al., 2011). As above, the different values that GPs hold and bring to consultations can contradict clinical practice guidelines, and in this sense, also shape the extent to which their understandings and approaches can be said to be pharmaceuticalised.

### Concluding Comments: A Spectrum of Pharmaceuticalised Understandings and Approaches

Looking across these themes, reflecting the ways in which knowledge, treatment perspectives and moral values were combined and drawn on by individual GPs, GPs arguably exist on a spectrum of how pharmaceuticalised they are in their approach to the primary prevention of CVD. Those on one end of the spectrum can be characterised as the most pharmaceuticalised in their understandings and approaches. These GPs were the most positive and emphasising of the benefits of statins within the ≥10% risk category. GPs towards this end of the spectrum saw the drugs as morally neutral, superior within the therapeutic landscape, and their widening usage the reflection of the best available evidence. These GPs were often also positive about guidelines and EBM more generally. There were no examples in the data of GPs critical of EBM but who viewed statins positively at the 10-19% risk level. On the other end of the spectrum, however, are those GPs that can be argued to be the least pharmaceuticalised in their approach. Indeed, one GP at the polar extreme was completely ignoring the 10%-19% threshold of risk, reporting that they did not necessarily conduct risk testing or discuss lifestyle changes with patients in this risk category. More generally, GPs at this end of the spectrum often reluctantly discussed with patients’ risk in this category, with a very heavy emphasis on making lifestyle changes instead of initiating a statin, and raised ethical (such as ‘needlessly’ worrying patients) and moral issues. Indeed, statins had the most significant moral baggage for this group of GPs—and emphasis was placed on not using drugs to treat problems of lifestyle. This group often were also the most emphasising of the importance of individual professional experience/knowledge and the most critical of EBM (see, for example, extracts from GP17). Distinctive from these GPs are those who are partially pharmaceuticalised in their understanding and approach, but often viewed lifestyle changes as necessary and/or importantly prior to initiating statins. In this regard, GPs in the middle of the spectrum displayed some concern about patients taking a statin without making changes where necessary to lifestyle behaviours on health and moral levels. However, GPs more in the centre of the spectrum often practiced closely to the guideline and also displayed trust in NICE. GPs in the centre of the spectrum were also often younger and/or less experienced professionals. Lesser clinical experience and the greater prominence of EBM within their medical education compared with older colleagues was likely to be important in practice being most clearly guided by the guideline.

It seems likely in a context of continuing trust, albeit conditional trust, in professionals (Calnan and Rowe, 2008) and muted consumerist tendencies in CVD prevention (Will and Weiner, 2015) that disparate professional approaches to and understandings about pharmaceuticalisation will have differential impacts on patient decision-making. Nevertheless, further research is required to establish the precise actualities of the impacts of differential pharmaceuticalised approaches to CVD prevention by GPs, and their positioning on the spectrum, in the configuration of patient understandings and identities relating to CVD prevention and taking statins. Where a patient and a GP hold (initially) distinct perspectives on pharmaceuticalisation it may be of particular analytical interest to establish how important the pharmaceuticalised approach of a GP positioned at the polar ends of the spectrum is in this context and the associated impacts on pharmaceuticalisation.

Overall, the disparate approaches evident in the data analysed suggest that GPs cannot be considered universally and uncritically to be a driving force of CVD pharmaceuticalisation within the ≥10% risk category. The value of this paper is that it provides clear indication of the disparate understandings of and approaches to pharmaceuticalisation displayed by GPs and the array of factors that feature in how they understand statins and report approaching decision-making about statins. This paper fills an important lacuna in knowledge about pharmaceuticalisation as conceptualised by Williams et al. (2011). In this regard, this paper has shown how even in the context of clinical practice guidelines recommending widening pharmaceuticalisation, GPs’ use of the knowledge embodied by guidelines is not uniform.

## Data Availability

The datasets presented in this article are not readily available because of the need to protect participant anonymity. Requests to access the datasets should be directed to t.douglass@ulster.ac.uk.

## Footnotes

1 However, see Will and Weiner (2015) for discussion of a failed attempt to make the drugs available over-the-counter.
